# Effect of Physical Activity and Obesity on Type 2 Diabetes in a Middle-Aged Population

**DOI:** 10.1155/2009/195285

**Published:** 2009-10-19

**Authors:** Rashid M. Ansari

**Affiliations:** School of Population Health, University of Queensland, Herston, QLD 4006, Australia

## Abstract

*Background*. The physical activity has been associated with a reduced risk of type 2 diabetes. The aim of this study was to examine the effect of physical activities such as occupational, household and daily lifestyle activities and obesity on the prevalence of type 2 diabetes in middle-aged population. *Methods*. All people (*n* = 2053), aged 45–64 years were selected for this study from the large sample of population-based cross-sectional data collected in the 1990–1994 by National Health Survey of Pakistan. The participants completed in-person interviews at baseline; the overall response rate was 92.6%. The Cox proportional hazards model was used to estimate the risk of developing the type 2 diabetes. *Results*. Stair climbing was found to be inversely associated with the risk of diabetes and cycling was also associated with a reduced risk of type 2 diabetes (RR = 0.82; 95% CI 0.68–1.00, *P* = .048). The relationship between physical activity and reduced risk of diabetes adjusted for age and body mass index was statistically significant only in women (*P* < .01). *Conclusions*. This study provides an incentive that physical activity in leisure-time exercise or daily activity reduces the risk of type 2 diabetes in a high-risk population.

## 1. Introduction

The objective of this article is to study the relationship of self-reported physical activity with obesity and diabetes prevalence in a middle-aged population of Pakistan. The middle-aged population of Pakistan is overweight or obese, lack of physical activity, unhealthy food, and eating habits exposing this population to a high risk of type 2 diabetes. According to World Health Organization (WHO), prevalence of Type 2 diabetes in Pakistan for the year 2000 was 5.2 million and for 2030 it would be around 13.8 million. A quarter of the population of Pakistan would be classified as overweight or obese with the use of Indo-Asian-specific BMI cutoff values. Jafar et al. [1] have reported that prevalence of overweight was 25% and obesity was 10% in a large population-based sample of people over the age of 15 years in Pakistan using revised definitions of overweight (BMI ≥ 23 kg/m^2^) and obesity (BMI ≥ 27 kg/m^2^) among Asian population [2]. This definition showed lower than the conventional cut off values of BMI (≥25 kg/m^2^) and obesity for populations of European origin and reflects the higher ratio of body fat to muscle mass. They have also used a cutoff BMI value of 23 kg/m^2^ in their data analysis using receiver operating characteristic (ROC) curve analysis for conditions such as hypertension and diabetes. On the age-specific prevalence of overweight and obesity, they found that more than 40% of women and 30% of men aged 35–54 years were classified as overweight or obese [1].

It has been suggested in a variety of observational and epidemiological studies that physical activity may play a significant role in the prevention of type 2 diabetes mellitus. The relationships between physical activity and overweight are only beginning to be understood for the adult population, sedentary behaviours, particularly watching television (TV) and videos as well as surfing the internet has been found to be related to higher body mass index (BMI) for adult's population [3]. Literature linking physical activity levels with risk of overweight in adults is not consistent but physical activity is an important component of effective obesity treatments [4].

The recent findings of the clinical trials in this area provide the most convincing evidence that physical activity, in conjunction with diet and weight loss, can prevent diabetes in a variety of populations and age groups [5–7]. More specific studies in men and women with impaired glucose tolerance at baseline from a variety of racial and ethnic backgrounds in the United States, China, and Finland demonstrated a decrease in the incidence of type 2 diabetes as the result of interventions that included physical activity [5–7]. However, with the exception of the Chinese study, which was randomized by clinic, all of these intervention trials combined physical activity with weight loss and diet in their intervention scheme. In other words, the independent effect of physical activity intervention was not tested directly.

The Word Health Organization conducted a survey in 2003 in Pakistan with a response rate of 95% and indicated that prevalence of physical inactivity is 26.5% among the males aged 50–59 years and 35% among females with the same age group.

The physical activity [8] can play an independent role in the prevention of type 2 diabetes separately from its effect on weight loss and body composition. The exercise and training studies have supported the contention that physical activity improves insulin sensitivity independently of any effect of activity on weight loss and fat distribution [8]. In a recent cross-sectional population study, physical activity was shown to be negatively associated with insulin concentrations in two populations at high risk for diabetes that differed greatly by body mass index [9].

The population in Pakistan has one of the world's highest documented incidence rates of type 2 diabetes mellitus, and has a high prevalence of obesity similar to those American Indians reported by Bennett et al. [10] and Knowler et al. [11]. Therefore, physical activity levels have been assessed in this population using the same activity questionnaire that is being used in a number of epidemiological studies of diabetes worldwide [12, 13]. The present study examined the role of physical activity and obesity in the development of type 2 diabetes risk in a cohort of middle-aged population in Pakistan.

## 2. Material and Methods

### 2.1. Study Description

This study used the specific data of middle-aged high risk population from the National Health Survey of Pakistan (NHSP) which was conducted between 1990 and 1994 by the Pakistan Medical Research Council (PMRC) and the Federal Bureau of Statistics (FBS), with technical assistance from the National Center of Health Statistics (NCHS) of the US Centers for Disease Control (CDC). The National Health Survey of Pakistan was a cross-sectional population-based survey, conducted between 1990 and 1994, of 18 135 individuals aged 6 months and above; 9442 of them were aged ≥15 years [14]. The sampling details, design, components, survey instruments and quality control have previously been reported [14]. Ethical approval for the survey was obtained from the Institutional Review Board at the Pakistan Medical Research Council [15].

All people (*n* = 2053), aged 45–64 years, were selected from the large sample of population for this study. The participants completed a detailed baseline survey including personal interviews for assessment of dietary intake, physical activity, and other lifestyle factors, and the overall response rate was 92.6%. The diabetes was diagnosed if the 2-hour elapsed time plasma glucose concentration was at least 200 mg/dL or if a diagnosis was conducted during the course of routine medical care [16]. The test was based on the standard 2-hour Oral Glucose Tolerance Test (OGTT) recommended by American Diabetes Association (ADA) as the standard 2 hour OGTT is sufficient to diagnose or exclude all forms of diabetes mellitus at all but the earliest stages of development. The examination was also conducted taking medical history, physical examination, and measurement of height and weight and obesity was estimated by the body mass index (weight (kg)/height (m)^2^). The category of overweight and obesity was defined as a BMI of 23 kg/m^2^ or greater. This definition, based on the revised criteria for Asian populations [2] was lower than the conventional cutoff value of 25 kg/m^2^ for populations of European origin. Obesity was defined as a BMI of 27 kg/m^2^ or greater. The measurements of weight, height, and circumferences of waist and hips were taken at baseline recruitment according to standard protocol by trained interviewers and from these measurements, the following variables were created: BMI: weight in kilogram divided by the square of height in meters, WHR: waist circumference divided by hip circumference.

### 2.2. Physical Activity

The detailed assessment of physical activity was obtained using a physical activity questionnaire which was administered by trained interviewers to individuals between the ages of 45 and 64 years who participated in the survey [14]. The validity of the questionnaire was evaluated by comparing Spearman correlations (*r*) for the physical activity questionnaire with two criterion measures administered over a period of 12 months. Significant correlations between the physical activity questionnaire and criterion measures for exercise were observed (physical activity log, *r* = 0.74; 7-day physical activity questionnaire, *r* = 0.80). Significant correlations between the physical activity questionnaire lifestyle activities and the 7-day questionnaire were also observed (*r* = 0.30–0.88). The reproducibility of the questionnaire (2-year test-retest) was evaluated using kappa statistics and intraclass correlation coefficients (kappa = 0.64 and the intraclass correlations coefficients were between 0.14 and 0.54).

The questionnaire evaluated regular exercise and sports participation during the last 2 years. All the participants were asked first if they had engaged in regular exercise/sports during the past 2 years. Exercisers were asked to report details for up to three types of exercises/sports (i.e., type, hours/week, and years of participation in each activity). Physical activity energy expenditure was estimated using standard metabolic equivalent values (MET) [17]. Exercise/sports energy expenditure was estimated by the weighted average of energy expended in all activities reported over the last 2 years preceding the (MET-hours/day/year). MET or the standard metabolic equivalent is a unit used to estimate the amount of oxygen used by the body during physical activity. 1 MET = the energy (oxygen) used by the body at rest, while sitting quietly or reading a book, for example. The harder the body works during the activity, the more oxygen is consumed and the higher the MET level.

The information was collected on daily activities such as walking, stair climbing, cycling and household activities. The metabolic equivalents for each daily activity and the total METs for daily activity were also calculated. Summary energy expenditure values (MET-hours/day) for these activities were estimated using the following MET values: housework, 2.0 METs; walking, 3.3 METs; stair climbing, 9.0; METs, and cycling 4.0 METs, using a compendium of physical activity values [17].

### 2.3. Statistical Analysis

The Cox proportional hazards model was used to estimate the risk of type 2 diabetes by levels of physical activity in the individuals who were nondiabetic at baseline. The physical activity and body mass index were included as time-dependent variables in these models, along with age at baseline. We used a quadratic term to adjust for age in the Cox proportional hazards model to allow for the nonlinear effect of age on the prevalence of diabetes. All analyses were performed separately for men and women.

The tests for linear trend were performed by entering the categorical variables as continuous parameters in the models. In all models, we adjusted for potential confounding variables including age, income level, education level, occupation, and smoking. We adjusted for pre-existing chronic disease (coronary heart disease, stroke, and cancer) and for hypertension to minimize the possibility that changes in physical activity following diagnosis of such conditions would obscure the true association between physical activity and prevalence of diabetes. In addition, the analyses were repeated after exclusion of participants with chronic diseases.

## 3. Results and Discussions

There were 482 cases of type 2 diabetes identified out of 2053 nondiabetic participants aged 45–64 years initially interviewed and were part of this research study. The participants with type 2 diabetes have a higher BMI, WHR, low level of education and had a history of CHD, hypertension and stroke as compared to nondiabetic women in the survey. Table 1 provides age-specific prevalence of obesity in middle-aged population of Pakistan. It may be observed that the prevalence is highest, 42% (95% CI = 38.3–46.1) among women aged 45–54 years and lowest, 26.7% (95% CI = 22.5–30.9) among men aged 55–64 years. Participants who engaged in leisure time physical activity were more likely to be older and to have a college education but less likely to be employed and participation in leisure time activity was also associated with having coronary heart disease, cancer, stroke, and hypertension. These results are in line with the findings of Jafer et al. [1] suggesting that that the factors independently and significantly associated with being overweight and obese in that population were higher age, being female, urban residence, and having a high income and high intake of meat. In addition, being overweight and obese was independently associated with having hypertension, diabetes, and raised serum cholesterol concentration [1].

Stair climbing was inversely associated with the risk of diabetes and this was true before and after the adjustment for BMI and WHR. Cycling was also associated with a reduced risk of type 2 diabetes (RR = 0.82; 95% CI 0.68–1.00, *P* = .048). Household activities and walking were unrelated to the risk of diabetes. When we examined the risk of diabetes by the quartiles of the distribution of METs from all daily activities combined, we found an inverse association of marginal significance (*P* = .055) before and after the adjustment for BMI and WHR.

The time-dependent Cox proportional hazards model was used to estimate the age-adjusted effect of physical activity on diabetes prevalence alone and then with body mass index added to the model as shown in Table 2. The total physical activity was significantly related to diabetes prevalence in women (*P* < .05) but not in men. If occupational activity is removed from the physical activity estimate, the relation between leisure physical activity and diabetes prevalence is strengthened, particularly in men. After adjustment for body mass index, the relation between activity and diabetes prevalence was weakened in both men and women. Similar results were found if weight was added to the model instead of body mass index. This relation between physical activity and diabetes prevalence adjusted for age and body mass index was statistically significant only in women (*P* < .01). There was no significant interaction between age, body mass index, or sex and total physical activity.

## 4. Strength and Weakness

In this study, the association between obesity and health conditions was cross-sectional and there was no follow-up study and therefore new cases of disease could not be identified. However, those could be considered in future efforts by more frequent assessment of physical activity during the follow-up period. Although some sociodemographic factors associated with being overweight have been identified in the survey, in-depth trials are needed to study the impact of strategies aimed at behaviour change that would target these factors to reduce the prevalence of being overweight or obese. Reliance on self-reported physical activity with obesity and diabetes is a major weakness of the study. Misclassification of diabetes could weaken the association between physical activity and the risk of type 2 diabetes. In addition, measurement error in the physical activity questionnaire may also introduce biases in the study. The strengths of this study are the high response rate and use of the modified Asian-specific definition of being overweight or obese which are generalizable. The other strength of this study is the high quality of data collected and the large sample size available for analysis allowing to assess the risk with adjustment for confounders for many demographic and socioeconomic factors as well as to evaluate potentially important interactions.

## 5. Conclusions

The main contribution of this study is to provide an incentive that physical activity in leisure-time exercise or other types of daily activities such as stair climbing, household activities, cycling, and walking reduce the risk of type 2 diabetes in a high-risk population. While the association between leisure-time and type 2 diabetes has been investigated in a number of studies [18–20], the influence of other types of physical activities on type 2 diabetes risk has not been systematically described.

This study has provided evidence that not only leisure-time physical activity but also other types of physical activities are associated with a reduction in type 2 diabetes risk. This study comprehensively evaluated the association of various types of physical activities and identified that stair climbing was inversely associated with the risk of diabetes and cycling was directly associated with a reduced risk of type 2 diabetes.

The finding that a physically active lifestyle is associated with a lower incidence of type 2 diabetes has been shown in several prospective studies. In a study of female-registered nurses aged 34–59 years at baseline, women who reported engaging in vigorous exercise at least once a week had a lower incidence of self-reported type 2 diabetes during the 8 years of follow-up than did women who did not exercise weekly [18]. Similar findings were observed between exercise and incidence of type 2 diabetes in a 5-year prospective study of male physicians aged 40–84 years [19]. Likewise, in a large cohort of postmenopausal women aged 55–69 years, the 12-year incidence of diabetes was lower in those women who reported any physical activity compared with the sedentary women [20]. In all of these prospective studies, however, the diagnosis of diabetes was based upon self-reported, physician-diagnosed diabetes. The results of the present study are consistent with the abovementioned prospective studies in literature [18–20].

In order to understand the potential contribution of physical activity to the prevention of type 2 diabetes apart from its effect on weight loss and body composition, one must define the physiologic basis underlying the relation between activity and diabetes. Beyond the effect of activity on body mass and composition, physical activity may reduce the risk for type 2 diabetes directly through improvements in insulin sensitivity [6]. However, a large portion of the effect of physical activity in decreasing insulin resistance is short lived and may last only a few days [21, 22]. Thus, the consistency of an individual's activity throughout the years is a key issue that needs to be measured before one can understand the mechanisms underlying the relation between physical activity and diabetes prevention [23].

The principle implications of this study is to highlight the importance of physical activity for the prevention of type 2 diabetes in the middle-aged population of Pakistan which is experiencing a rapid and substantial decline of physical activity levels as a result of poor eating habits, unhealthy food supply, expansion of television, computerization, and mechanization as well as more prevalent car ownership and sedentary behaviour. In parallel with decreasing levels of physical activity, the prevalence of overweight and obesity has increased significantly in Pakistan and as a consequence, diabetes mellitus has become a major public health issue. Therefore, promoting an active lifestyle or regular exercise should receive the highest public health priority in that country.

## Figures and Tables

**Table 1:** Age-specific prevalence of obesity in middle-aged population (*n* = 2053).

Age, years	Obese women	% (95% CI)	Obese men	% (95% CI)
45–54	263/623	42.0 (38.3–46.1)	161/559	28.8 (25.1–32.6)
55–64	140/444	31.5 (27.2–35.9)	114/427	26.7 (22.5–30.9)

Total	403/1067	37.7 (34.9–40.7)	275/986	27.9 (25.1–30.7)

**Table 2:** Time-dependent proportional hazard regression predicting diabetes. Incidence by physical activity level in the middle-aged population.

	Population/total sample	Variable	Hazard ratio	95% confidence interval	*P* value
Men	275/986				
	Controlled for age	Leisure activity	0.60	0.40–0.90	.05
		Total activity	0.79	0.46–1.25	.5
	Controlled for age and body mass index	Leisure activity	0.78	0.57–1.28	.06
		Total activity	1.0	0.60–1.58	.8

Women	403/1067				
	Controlled for age	Leisure activity	0.68	0.50–0.92	.01
		Total activity	0.70	0.53–0.95	.04
	Controlled for age and body mass index	Leisure activity	0.72	0.58–0.97	.04
		Total activity	0.75	0.65–1.08	.08
